# Reply to Sarridou et al.

**DOI:** 10.1007/s00540-021-02964-2

**Published:** 2021-07-16

**Authors:** Andreas Koster, Nikolai Hulde, Armin Zittermann

**Affiliations:** 1 Institute for Anesthesiology and Pain Therapy, Herz-Und Diabeteszentrum NRW, Ruhr-University Bochum, Bad Oeynhausen, Germany; 2 Clinic for Thoracic and Cardiovascular Surgery, Herz-Und Diabeteszentrum NRW, Ruhr University Bochum, Georgstrasse 11, 32545 Bad Oeynhausen, Germany

**Keywords:** Neurotoxicity, Renal dysfunction, Tranexamic acid, Etomidate

To the Editor:

We appreciate the comments by Sarridou et al. [1] regarding our article [2], since they highlight the importance of “drug safety” in anesthesia. With respect to etomidate-induced convulsive seizures (CS), it is noteworthy that they occasionally appear immediately after administration of the induction dose, persist for only a few minutes [3], and are not routinely assessed. Tranexamic acid (TXA)—associated CS, however, are usually observed in the intensive care unit, at a time when TXA concentrations in the cerebral fluid reach a maximum [4]. Since etomidate is associated with more stable hemodynamics than its competitors without increasing adverse outcomes [5], it is difficult to replace etomidate as an induction agent in cardiac surgery.

We agree with Sarridou et al. that our study was probably underpowered for the detection of an interaction between history of stroke and risk of CS. Nevertheless, it is also important to note that even in our large cohort of cardiac surgical patients no statistical evidence could be provided that history of stroke is a contraindication for TXA use.

The effect of renal impairment on the TXA-induced risk of CS is indeed well-known and in patients with chronic kidney disease even a single bolus dose of 1 g TXA (equivalent to about 10–15 mg/kg body weight) was associated with a higher risk of CS than in patients without renal impairment [6]. However, this latter data are limited by the small number of patients with chronic kidney disease and further supportive studies are thus required. A recent meta-analysis on the efficacy and safety of TXA, which focused on CS for safety, suggested a cumulative dose of approximately 15 mg/kg body weight for a bolus plus continuous infusion regimen and 20 mg/kg body weight for a bolus-only regimen [7]. In our opinion, these are values that should be commonly considered in clinical practice.
